# Longitudinal Analysis of Sweet Taste Preference Through Genetic and Phenotypic Data Integration

**DOI:** 10.3390/foods13213370

**Published:** 2024-10-23

**Authors:** Ji Hyun Bae, Hyunju Kang

**Affiliations:** Department of Food Science and Nutrition, Keimyung University, Daegu 42601, Republic of Korea; jhb@kmu.ac.kr

**Keywords:** sweet taste preference, obesity, personalized dietary recommendations, longitudinal analysis

## Abstract

Understanding the genetic basis of sweet taste preference is crucial for potential implications in diet-related health outcomes, such as obesity. This study identified genes and single nucleotide polymorphisms (SNPs) associated with sweet taste preferences over time. Data from the American Nurses’ Health Study (NHS1) and Health Professionals Follow-up Study (HPFS) cohorts were analyzed. Using tools like PLINK and METAL for genetic associations and FUMA for functional annotation, the study identified eight SNPs associated with sweet taste preferences. Notably, rs80115239 and rs12878143 were identified as key determinants of the highest and lowest associations with sweet taste preferences, respectively. Individuals with the rs80115239 (AA) genotype displayed a higher preference for sweet tastes, including chocolate and cake, but a lower preference for physical activity, fruits, and vegetables, particularly in females from the NHS1 cohort, linking this genotype to a higher obesity risk. Conversely, those with the rs12878143 (CC) genotype preferred fruits, vegetables, coffee, and tea, with a lower preference for sweetened beverages, but the correlation with obesity risk was less clear due to inconsistent data. In conclusion, these findings highlight the genetic influences on sweet taste preference and their potential role in personalized dietary recommendations and obesity management strategies.

## 1. Introduction

The complex interplay of environmental and genetic factors plays a pivotal role in shaping our dietary choices and consumption levels of sweet foods. These choices bear critical nutritional and health implications, influencing the risk of obesity, type 2 diabetes, cardiometabolic diseases, and metabolic syndrome [1,2]. Taste perception and preferences, which are heritable traits [3], serve as significant determinants in food choice and consumption. Food preferences are not solely dictated by culture and familiarity but also have a strong biological foundation [4]. For instance, variations in sweet taste receptors such as taste 1 receptor member 2 (TAS1R2) have been linked to dietary sugar intake [5], underscoring the genetic influences on taste preferences [6]. While some inconsistencies exist in the relationship between sweet taste perception and actual food intake [7], a strong correlation has been observed between sweet taste preference and the consumption of sugary foods [8].

In humans, Type 1 and Type 2 taste receptors (T1R and T2R) are responsible for the perceptions of sweet and umami tastes, and bitter tastes, respectively [9]. Common polymorphisms in *TAS2R38*, a gene encoding a member of the T2R family, have been notably associated with bitter taste perception [10]. Taste transduction and processing are also influenced by these polymorphisms [6,11]. Similarly, the genetic influence on sweet taste perception at the population level has been linked to specific receptor subunits and pathways [12,13]. The increasing interest in the factors driving food consumption is aimed at directing consumers towards choices that reduce the burden of various diseases. Food consumption has been reported to correlate with numerous factors, including personal preference [4,14]. Therefore, predicting or measuring food preference can be a crucial first step in designing more effective, targeted dietary interventions and creating more acceptable, nutritious foods. Food preference is a moderately heritable trait influenced by genetic factors. In children, it is shaped by shared environmental effects [15], whereas in adults, it is largely influenced by personal experience [16]. A prime example of these dynamics is evident in the consumption of sweet-tasting foods, which are widely consumed and have a distinct flavor profile that transcends mere sweetness. Naturally sweet-tasting foods typically contain a range of bioactive compounds, including flavonoids, which have been linked to various health benefits and risks [17]. This contrasts with processed sweet foods, where preferences and consumption patterns can be markedly different [14]. Studying sweet taste preferences, particularly in relation to foods like chocolate, ice cream, and cake, offers unique opportunities compared to other foods for informing public health strategies and targeted nutritional interventions.

Longitudinal studies have been instrumental in identifying common genetic variants that mediate taste perception and preference, thereby elucidating the genetic underpinnings of dietary choice and consumption over time. While previous research has identified genes associated with bitter taste [18], similar studies focusing on sweet taste are noticeably scarce [19,20]. Some progress has been made, such as the identification of a lead single nucleotide polymorphism (SNP) in the *PTPRN2* gene that is associated with sweet taste preference and, consequently, the intake of sugary foods [21]. Recently, it was reported that sweetness-preference-related polymorphisms were associated with sex-dependent variations in obesity [13]. However, challenges such as limited cohort sizes and phenotypic heterogeneity have hampered efforts to identify genes related to sweet taste preferences, including chocolate, ice cream, and cake [22,23]. 

In light of these limitations and gaps in current understanding, the present study aimed to delve into a longitudinal analysis of sweet taste preferences. By integrating genetic and phenotypic data, we aimed to enhance our understanding of the genetic factors contributing to the relationship between taste preferences and dietary intake of sweet-tasting foods. Furthermore, we explored associations among various taste preferences, hypothesizing that a sweet taste preference may not only be influenced by genetic factors but also be associated with other taste preferences, such as preferences for smoking, coffee, or tea. Additionally, we explored whether these associations could potentially impact risk factors for obesity and other health conditions and whether the correlations might depend on sex, to inform personalized dietary recommendations and targeted obesity management.

## 2. Materials and Methods

### 2.1. Study Populations

**Figure 1:**
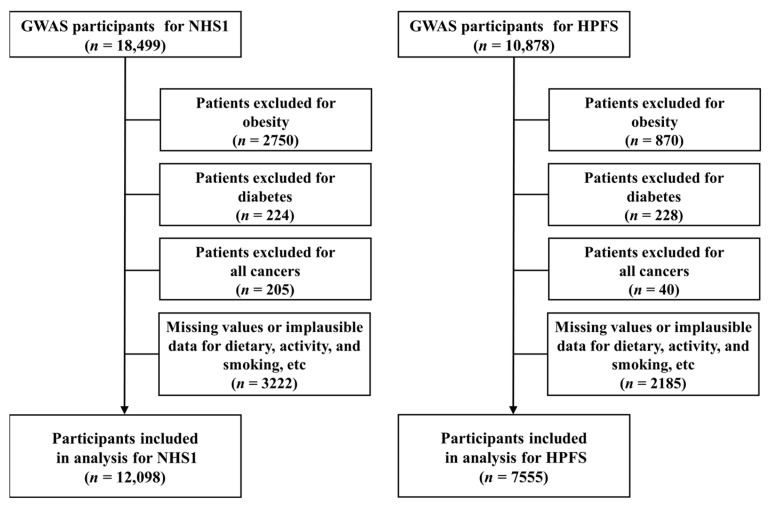
Schematic diagram of the simplified process for selecting study subjects. *n*, number of subjects.

The study population consisted of two large prospective cohorts: the American Nurses’ Health Study (NHS1) and the Health Professionals Follow-up Study (HPFS) cohorts. The NHS1 cohort was established in 1976 when 121,701 female registered nurses, aged 30–55 years, completed self-administered questionnaires at the time of cohort inception. The HPFS cohort included 51,529 males, aged 40–75 years, at the time of enrollment in 1986. Participants in both cohorts were followed up with biennial questionnaires regarding their medical histories and lifestyles, and semi-quantitative food-frequency questionnaires (FFQs) were completed every 4 years. In this study, the baseline year for both cohorts was 1986, when detailed information regarding participants’ dietary habits and lifestyles was available. Regarding blood samples, 32,826 females in the NHS1 cohort provided samples between 1989 and 1990, while 18,225 males in the HPFS cohort provided samples between 1993 and 1995. With both cohorts, DNA was extracted from white blood cells using the QIAamp Kit (QIAGEN, Germantown, MD, USA) and the manufacturer’s protocol for blood samples, all of which were processed at the same laboratory [24]. This study included 18,499 females and 10,878 males of European ancestry who had complete baseline information and available genotype data for genome-wide association studies (GWASs) [25,26]. All participants were free from diabetes and cancer at the baseline. We also excluded females and males with a BMI (in kg/m^2^) > 30 at the baseline since individuals in this category may possess distinct metabolic profiles or genetic predispositions that could influence taste preferences or the impact of specific diets on health outcomes. Additionally, we excluded individuals with missing or implausible FFQ responses. Following exclusion based on these criteria, the final cohort used for data analysis included 12,098 females and 7555 males, as shown in Figure 1. 

This study was approved by the institutional review board of Keimyung University (approval number 40525-202110-BR-066-01). The study protocol was approved by the institutional review boards (IRBs) of the Brigham and Women’s Hospital and the Harvard T.H. Chan School of Public Health, and the IRBs allowed participants’ completion of questionnaires to be considered as implied consent.

### 2.2. Assessment of Obesity and Covariates

Height and body weight data were self-reported in questionnaires administered at enrollment and each follow-up. Weights reported in questionnaires versus technician-based anthropometric measurements were highly correlated (Pearson correlation coefficient [r] = 0.97 in both studies) in a validation subsample [27]. The BMI was calculated in terms of kg/m^2^, and subjects with a BMI greater than 30 kg/m^2^ were classified as obese. The subjects’ age, ethnicity, smoking status, and level of physical activity were obtained from the biennial questionnaires. We converted the average time spent per week participating in physical activities (e.g., walking, running, and biking) to metabolic equivalent hours (METs) per week [28]. The reproducibility of the physical activity data had been validated previously [29]. Alcohol intake was assessed on the FFQ every 4 years, and trans-fat and total energy intake were updated from these questionnaires. Age was calculated based on the date of birth and the date the questionnaire was returned.

### 2.3. Dietary Assessment and Phenotype Definitions

Participants were asked about their preferences and frequencies of consuming specific foods and beverages during the past year, and the participants’ food intake frequencies were quantified using nine categories: “never/almost never”, “one to three times a month”, “once a week”, “two to four times a week”, “five to six times a week”, “once a day”, “two to three times a day”, “four to five times a day”, and “more than six times a day” [30]. Sweet taste preference was classified into two groups by calculating the cumulative average sum intake of sweet foods such as chocolate, cookies, brownies, donuts, cake, and jam. Despite the variation in taste profiles among these foods, from the bittersweet nuances of chocolate and brownies to the purely sweet flavors of jam and cake, this methodological decision to group them together was based on the premise that their collective consumption might reflect an overarching sweet taste preference. In this manner, two groups were created: “low” sweet taste preference (less than five servings per month) and “high” sweet taste preference (more than 50 servings per month) to determine the sweet taste preference phenotype [31]. 

### 2.4. DNA Isolation and Quality Control

In the NHS cohort, 32,826 blood samples and 29,684 cheek cell samples have been collected since the late 1980s. In the HPFS, blood samples from 18,159 participants and cheek cell samples from an additional 13,956 men have been collected. The DNA extracted from white blood cells and cheek cells has been crucial for conducting GWAS, sequencing, and evaluating telomere length, as previously described [32]. 

### 2.5. Genotyping

A GWAS was performed to identify novel loci that may influence the sweet taste preference. We used a pooled GWAS dataset comprising 29,377 participants, including 18,499 females and 10,878 males of European ancestry, from the NHS1 and HPFS cohorts, respectively. Genotyping and merging were performed with the pooled GWAS dataset, which was generated using five different platforms (Illumina HumanHap arrays, Illumina OmniExpress arrays, Humancore array, Oncoarray, and Affymetrix 6.0 array), as described in detail by Lindström et al. [32]. The individual datasets genotyped on the same platform were combined, and SNPs with duplicate identification numbers were excluded. The Hardy–Weinberg equilibrium, imputation quality, and other quality control filters were independently applied for the samples and SNPs in each GWAS. Samples with a missing call rate > 5% (with any platform) during the merging process were excluded from further analysis. SNPs with a minor allele frequency < 1% or an imputation quality (r^2^) < 0.5 were also excluded [33]. Missing genotypes were imputed using the Haplotype Reference Consortium as the reference panel [34]. 

### 2.6. Functional Annotation and Gene Mapping

Functional annotation was performed using Functional Mapping and the Annotation of Genome-Wide Association Studies (FUMA GWAS, an online platform for the functional mapping of genetic variants), and genomic risk loci were defined by merging lead SNPs that physically overlapped or for which linkage disequilibrium blocks were less than 250 kb apart [35]. In addition, Combined Annotation Dependent Depletion (CADD) [36] scores (with scores > 12.37 indicating the deleteriousness of an SNP) and RegulomeDB [37] scores (with a lower score indicating a higher probability of having a regulatory function) were annotated to SNPs by matching chromosomal positions, reference alleles, and alternative alleles. SNPs in genomic risk loci that showed genome-wide significance were mapped to genes in the FUMA GWAS using one of two methods. First, positional mapping was performed based on the physical distances (i.e., within 10 kb windows) from known protein-coding genes in the human reference assembly (GRCh37 or hg19) to map SNPs to genes. The second method, expression quantitative trait locus (eQTL) mapping, was based on information from the GTEx data repository and was used to map SNPs to genes based on a significant eQTL association, where the gene-expression level is associated with allelic variation at the SNP locus [38]. The nearest gene to each SNP was identified by HaploReg v4.1 (Broad Institute, Cambridge, MA, USA), a tool for exploring annotations of the noncoding genome at variants on haplotype blocks, using the RefSeq genes [39].

### 2.7. Statistical Analysis

PLINK was used to conduct genome-wide association analyses [40,41] and to apply a logistic regression model to the two pooled GWAS datasets. A meta-analysis was conducted using the METAL software (University of Michigan, Ann Arbor, MI, USA) [42], and Cochran’s Q statistic was used to test for heterogeneity between females and males. Cox proportional hazard modeling with time-varying covariates was used to estimate age-adjusted and multivariable hazard ratios (HRs) and 95% confidence intervals (CIs) for incident obesity. All *p*-values were 2-sided, and a *p*-value < 0.05 was considered to indicate a statistically significant difference. Each participant’s follow-up time was calculated from the return date of the baseline questionnaire (i.e., 1986 for the NHS and HPFS) to the date of obesity diagnosis (BMI > 30 kg/m^2^), death, or the end of follow-up (June 2014 for the NHS1 cohort and January 2014 for the HPFS cohort), whichever occurred first [43]. Participants who reported a BMI > 30 kg/m^2^, cancer, or diabetes, or who died were excluded from subsequent follow-up. In addition to analyses adjusted solely for age, we developed a comprehensive multivariable model that adjusted for various factors, including age, smoking status, alcohol consumption, physical activity level, trans-fat intake, total energy, and total fiber intake. Among the covariates, age, physical activity level, trans-fat, total fiber, and total energy intake were included as continuous variables, while smoking status and alcohol consumption were treated as categorical variables. Adjusted multivariable HRs for both cohorts were pooled using fixed-effect meta-analysis with either SAS software (version 9.3; SAS Institute Inc., Cary, NC, USA) or R software (version 4.0.0; R Foundation for Statistical Computing, Vienna, Austria) [44]. 

## 3. Results

### 3.1. Longitudinal Analysis of SNP Associations with Sweet Taste Preference

**Figure 2:**
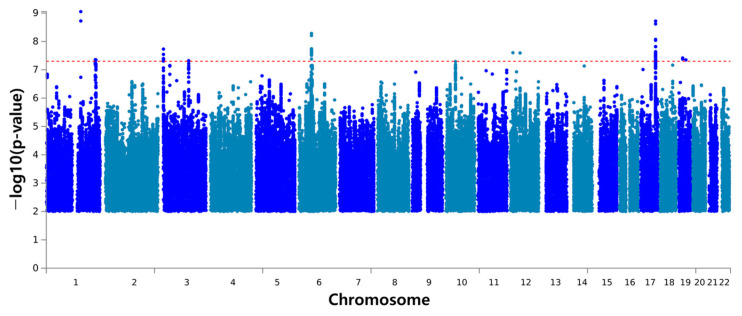
Manhattan plot for the sweet taste preference phenotype from a GWAS. The −log10 (*p*-value) of SNPs (*y*-axis) are plotted against their chromosomal positions (*x*-axis). Each dot represents a SNP, with the red dashed line indicating the *p*-value threshold for statistical significance. The most significant SNP on chromosome 1 is rs80115239 (*p* = 8.87 × 10^−8^), and the strongest association on chromosome 14 is rs12878143 (*p* = 7.38 × 10^−6^).

In our longitudinal study focusing on sweet taste preferences over time, analyses were conducted to identify genetic factors that may influence these preferences. Using logistic regression models, we analyzed SNPs based on a dichotomous variable of high or low sweet taste preference. Figure 2 presents the Manhattan plot, where eight SNPs emerged as significantly associated with sweet taste preference at a suggestive *p*-value (*p* < 1 × 10^−5^) for significance [21]. Unlike the stricter *p*-value threshold (*p* < 1 × 10^−8^) commonly used in disease-oriented GWAS, this approach reflects the understanding that the effects of genetics on dietary behavior tend to have subtle effect sizes and are distributed across a broad genetic landscape. These SNPs were rs80115239, rs7549176, rs73159751, rs10845710, rs2218442, rs12878143, rs58082448, and rs12979148.

Of these, two lead SNPs, rs80115239 and rs12878143, were selected for further investigation based on their notable genetic characteristics and implications. SNP rs80115239 stands out with a highly significant *p*-value of 8.87 × 10^−8^ and a low minor allele frequency (MAF) of 0.02, making it a strong candidate for influencing sweet taste preference. The other SNP, rs12878143, though with a relatively higher *p*-value of 7.38 × 10^−6^, has a notably low MAF of 0.06 compared to others, still indicating significant relevance. This is because their low MAF values are sufficient for analysis, underlining the variants’ significance [33]. When examining the odds ratios (OR), rs80115239 had the highest OR value of 1.34 (95% CI 1.20–1.49), supporting its positive association with sweet taste preference. On the other hand, rs12878143 had the lowest OR value of 0.84 (95% CI 0.77–0.90), suggesting a weaker association with sweet taste preference, which may be linked to aversion. The heterogeneity (I^2^) for both SNPs was zero, confirming that there was no heterogeneity between the two cohorts. Regarding the effect alleles, for rs80115239, the effect allele was “A”, with “G” being the non-effect allele. For rs12878143, “C” was the effect allele, and “T” was the non-effect allele. These parameters, including the odds ratios of our selected lead SNPs, strongly suggest that they are associated with the highest and the lowest preferences for the sweet taste, respectively. 

**Figure 3:**
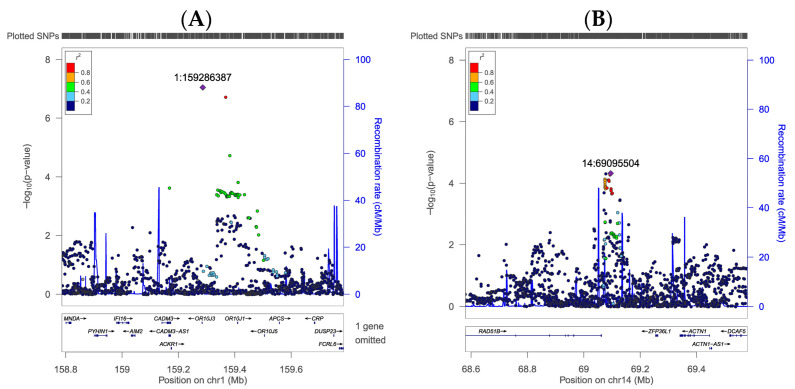
Regional association plot for the reference SNPs. (**A**) Regional association plot for the reference SNP rs80115239. SNPs are plotted by displaying the negative logarithm of the associated *p*-values as a function of the genomic position. SNP rs80115239 is located on chromosome 1 at nucleotide position 159286387 (based on GRCh37) and was identified as the most strongly associated SNP in chromosome 1 (rs80115239, purple diamond). (**B**) Regional association plot for the reference SNP rs12878143. SNPs are plotted by displaying the negative logarithm of the associated *p*-value as a function of the genomic position. SNP rs12878143 is located on chromosome 14 at nucleotide position 69095504 (based on GRCh37) and was identified as the most strongly associated SNP on chromosome 14 (rs12878143, purple diamond).

Additionally, we mapped the genomic locations of these SNPs. rs80115239 is located on chromosome 1, adjacent to the OR10J3 gene (Figure 3A), whereas rs12878143 is situated on chromosome 14, adjacent to the RAD51B gene (Figure 3B). The adjacent genes, OR10J3 for rs80115239 and RAD51B for rs12878143, might play roles in sensory perception and DNA repair mechanisms, respectively, potentially affecting taste preferences [45,46]. The specific positions in the human genome (hg19 build) were 1:159286387 for rs80115239 and 14:69095504 for rs12878143, respectively. More detailed information on the selected eight SNPs for the sweet taste preference is listed in Table 1, including the position, OR, *p*-value, corresponding MAF, and the annotated gene for the SNPs. The results showed that the selected lead SNPs, rs80115239 and rs12878143, are the SNPs most strongly associated with sweet taste preference, representing the highest and lowest associations, respectively, at a genome-wide level.

### 3.2. Relations of Sweet Taste Preference with Other Tastes

To examine the correlations between genetics and dietary habits, we conducted a focused analysis of the relationship between sweet taste preferences and other lifestyle factors, such as smoking, alcohol intake, and consumption of coffee, tea, or other foods. This analysis was facilitated by the identification of two lead SNPs, rs80115239 and rs12878143, which were evaluated for significant associations with sweet taste preferences using logistic regression models. We integrated age-standardized characteristics from two large-scale studies: the female NHS1 cohort and the male HPFS cohort, as presented in Table 2. The analysis revealed that individuals with the AA genotype displayed a higher preference for non-smoking and alcohol intake in NHS1 but not in the HPFS cohort. Moreover, this genotype was associated with an elevated preference for sweet tastes, including chocolate and cake, but a lower preference for physical activity, fruit, vegetables, coffee, and tea, compared to those with the GG or GA genotypes in the female NHS1 cohort. Individuals with the GA genotype did not show significant differences in NHS1. In HPFS, individuals with the AA genotype had a higher preference for sweetened beverages but a lower preference for fruit, vegetables, coffee, and tea compared to other genotypes. Individuals with the GA genotype in HPFS exhibited a higher preference for current smoking, alcohol intake, and the consumption of coffee, chocolate, ice cream, and cake. 

On the other hand, for rs12878143, Table 3 showed that individuals with the CC genotype had a higher preference for fruit, vegetable, coffee, and tea consumption but a lower preference for sweetened beverages compared to other genotypes in the female NHS1 cohort. In the male HPFS cohort, individuals with the CC genotype showed a higher preference for coffee consumption but a lower preference for sweetened beverages, ice cream, and cake. Individuals with the CT genotype exhibited a relatively lower preference for fruit, vegetable, coffee, tea, chocolate, and ice cream consumption in NHS1. However, the results indicated that these genotypes were not strongly associated with sweet taste preference due to inconsistent trends in sweet taste preferences. The scattered results could be attributed to the relatively higher CADD and RDB values (Table 1). Collectively, our analysis highlighted that rs80115239 was positively correlated with a variety of lifestyle preferences, such as non-smoking and alcohol consumption, in addition to sweet taste preference in the female NHS1 cohort. Although rs12878143 showed a higher preference for coffee consumption, it was not strongly associated with sweet taste preference and did not correlate with other dietary habits in the female NHS1 cohort. However, in the male HPFS cohort, this genotype was correlated with a lower interest in sweet taste preference, a finding that warrants further investigation in future studies. 

### 3.3. Relations of Sweet Taste Preference with Obesity

To explore the relationship between the identified lead SNPs and obesity in the context of sweet taste preference as a phenotype, we employed two statistical models: an age-adjusted model (Model 1) and a multivariate-adjusted model (Model 2). These models were applied to genotypic data from two cohorts: females from NHS1 and males from HPFS. In Table 4, for rs80115239 (AA vs. GG), the HR values were 1.822 with a 95% CI above 1.0 and a *p*-value of 0.004 in Model 1, and 1.902 with 95% CI above 1.0 and a *p*-value of 0.002 in Model 2 in the NHS1 cohort. Additionally, for this phenotype, the HR values were 1.642 in Model 1 and 1.716 in Model 2, both with reasonable 95% CI values above 1.0 and sufficiently low *p*-values in the pooled cohort. These findings imply that the phenotype exhibits a close association with obesity in the female NHS1 and pooled cohorts. In contrast, the HR values were close to 1.0 with questionable 95% CI and *p*-value for this phenotype in the male HPFS cohort, indicating a negligible correlation with obesity. For rs80115239 (GA vs. GG), the HR values were around 1.0 with non-significant 95% CI and *p*-value in both Models 1 and 2 across all cohorts considered, implying that this phenotype is not correlated with obesity. 

For rs12878143 (CC vs. TT) in Table 4, the HR values were 1.346 (95% CI: 1.083 -1.674; *p*-value: 0.007) in Model 1 and 1.347 (95% CI: 1.083–1.675; *p*-value: 0.007) in Model 2 in the female NHS1 cohort. Similar results were obtained in the pooled cohort, indicating a positive association with obesity. However, in the HPFS cohort, the 95% CI ranges were 0.869–1.791 in Model 1 and 0.870–1.792 in Model 2, which did not confirm the outcome. Additionally, these results were not consistent with previous findings that suggested this phenotype was weakly associated with sweet taste preference. Although the HR values for rs12878143 (CT vs. TT) were less than 1.0, suggesting a negative association with obesity in Models 1 and 2 across all cohorts studied, the 95% CI values and *p*-values were not sufficient to indicate a significant negative association with obesity risk. 

In summary, our analysis revealed a differential impact of the lead SNPs on obesity risk, depending on their association with sweet taste preference. Specifically, SNP rs80115239, which was associated with sweet taste preference, also showed a positive association with obesity in the female NHS1 cohort. In contrast, SNP rs12878143, which was less dependent on sweet taste preference, did not exhibit an association with obesity. These findings further suggest that genetic factors influencing sweet taste preference could also be linked to obesity risk.

## 4. Discussion

Sweet-tasting foods such as chocolate, ice cream, cake, and sweetened beverages, frequently consumed in the human diet, have been subjects of growing interest, not just for their sensory appeal but also for their potential health implications. We have uncovered compelling evidence that sweet taste preference is not merely a matter of personal choice but has a genetic basis, influencing the dietary intake of sweet-tasting foods as well as preferences for smoking and coffee consumption. Sweet taste perception and preference can be heritable determinants of food choice and consumption [3]. However, the specific genes and genetic variants that mediate sweet taste preference have largely remained unexplored. We investigated the correlation between genes and sweet taste preference using an SNP-based approach. Two SNPs, rs80115239 and rs12878143, were identified at a genome-wide significance level, showing differential associations with high or low preferences for sweet taste. Intriguingly, the variant rs80115239 was not only related to a higher preference for sweet taste but also showed strong associations with non-smoking and coffee consumption. Remarkably, we found a robust association between genetic variants and sweet taste preference and intake, which depends on sex. Specifically, we identified two key SNPs: one (rs80115239) positively related to sweet taste preference and another (rs12878143) hardly related to it, providing fresh insights into human sweet taste perception [19]. The SCAF11 SNP rs2218442 could also be a candidate as a lead SNP negatively associated with sweet taste preference, as its OR, MAF, and *p*-values are similar to those of rs12878143. However, it was noted that SNP rs2218442 is associated with the expression of genes in the artery tibial thyroid based on eQTL analysis; thus, we chose rs12878143 alone for presumed negative sweet taste preference in this analysis. 

Regarding the adjacent genes, OR10J3, associated with rs80115239, has been reported to be associated with monocyte chemoattractant protein 1 (MCP-1) in other regions of chromosome 1 [47]. Elevated MCP-1 levels are known to be present in chronic inflammatory diseases, including obesity [48], indicating a potential association between the OR10J3 gene and obesity. Additionally, the OR10J3 gene has been linked to tumor necrosis factor (TNF) [49], which induces inflammation related to obesity. Another adjacent gene, RAD51B, associated with rs12878143, is a protein involved in DNA repair and regulates cell-cycle-related processes, potentially affecting taste preferences [45].

Our findings also corroborate the idea that genetic variants influencing food choice can extend their effects to other taste perceptions and preferences [21]. In this context, these variants can significantly influence personalized nutrition strategies [50,51]. Consistently, the analysis of age-standardized characteristics according to the genotype of SNP rs80115239 showed that these genetic variants influence not only sweet taste preference but also other behaviors, such as smoking and the consumption of alcohol, coffee, and tea. Notably, there appeared to be a particular affinity between non-smoking and alcohol intake and a sweet taste preference for the SNP rs80115239 (AA) genotype compared with other phenotypes. Additionally, this genotype was correlated with reduced consumption of fruit, vegetables, coffee, and tea in both the NHS1 and HPFS cohorts. The findings of this study align with previous research, which suggests that genetic variants associated with alcohol consumption are predominantly related to sweet taste preference [31]. Given that these genetic variants influence a broad range of taste preferences, individuals with a genetic predisposition for enjoying sweet tastes are likely to consume not only sweet beverages but also sweet foods, with a preference for chocolate and cake [52,53]. On the other hand, SNP rs12878143 exhibited a distinct pattern. These variants were predominantly related to a preference for vegetables, coffee, and tea, correlating with a reduced consumption of sweet-tasting foods. Interestingly, the rs80115239 (AA) genotype, related to a preference for sweet tastes, was more likely to align with lifestyle phenotypic factors such as reduced consumption of vegetables, coffee, and tea. However, the rs12878143 (CC) genotype, which is less correlated with sweet taste, showed a stronger association with the consumption of vegetables, coffee, and tea. However, these specific genetic variants (rs80115239 and rs12878143) did not show a strong relationship with alcohol preferences within the scope of this study. Despite attempting to replicate findings from other studies that suggest a connection between sweet taste preference and alcohol intake, the relationship in this study was only marginal. 

We aimed to evaluate the gene-diet correlations related to sweet taste preferences and their connection to obesity risk. The adjusted HR values greater than 1.0 for the rs80115239 (AA) genotype in relation to obesity indicated that individuals with this genotype had a positive association with obesity. Given that the rs80115239 (AA) genotype is associated with a preference for sweet tastes and the consumption of sweetened beverages, chocolate, ice cream, and cake, individuals with this genotype are likely to have a higher total energy intake, potentially increasing obesity risk. In contrast, although the rs12878143 (CC) genotype was identified as being the least associated with or as showing disinterest in sweet taste preference, the data related to obesity were too scattered to determine its effects on obesity. Notably, the differential impact of the lead SNPs associated with sweet taste preference on obesity risk likely depends on sex. Specifically, SNP rs80115239, which was associated with sweet taste preference, also showed a positive association with obesity in females in the NHS1 cohort but not in males in the HPFS cohorts. 

Genetic variations related to taste preference can increase the risk of diet-related diseases such as obesity and diabetes. Significant associations have been identified between sweetness preference-related SNPs and both sweetness preference and obesity [13]. The gene encoding protein tyrosine phosphatase receptor type O (PTPRO), which is upregulated in adipose tissue due to obesity [54] and obesity-induced inflammation [55], has been strongly associated with sweetness preference. Since PTPRO is involved in the regulation of glucose and lipid metabolism and the inactivation of the insulin receptor [56], this further suggests a positive correlation between sweetness preference and obesity risk. Moreover, sweet taste receptors have been found to play a role in glucose absorption and metabolism by stimulating the release of glucagon-like peptide-1 (GLP-1) [57], which contributes to the pathological processes leading to diabetes [58]. Additionally, the TAS2R38 gene, which mediates bitter taste perception, may encourage the consumption of healthy dietary components such as fiber and vitamins by promoting the selection of vegetables and other nutrient-rich foods [59]. High sensitivity to bitter taste, however, can limit the consumption of these healthy foods, resulting in higher consumption of sweet-tasting substitutes, thereby elevating the risk of obesity [60]. Nevertheless, other contributory factors between the sweet and bitter taste receptor genes remain to be explored in future studies.

It is worth mentioning that while our study reveals compelling trends, there are areas that warrant further research. For instance, the relationship between sweet taste preference and alcohol intake remains marginally associated, requiring further exploration. Additionally, food insecurity has been reported to be positively associated with the risk of obesity in girls but negatively associated in boys [61]. Thus, the impact of gender on the relationship between these genetic variants and obesity risk warrants further investigation in future studies. Considering the average daily chocolate intake in the United States is relatively minor—about 14 g or roughly 75 calories, a small portion of a 2000-calorie diet [62]—this study faces a limitation in asserting sweet taste consumption as a significant contributor to obesity. The strong statistical associations, along with potential biological relevance, make rs80115239 and rs12878143 compelling subjects for further genetic and functional studies on sweet taste preference. Future research is warranted to explore these complex correlations and their implications on public health narratives and dietary guidelines. 

A limitation of this study is the unavoidable measurement errors associated with self-reported data. The inherent bias in self-reporting required that participants be well-trained to accurately use the devices involved in the data collection process. Indirect estimation of BMI is also a limitation, as individuals with different body proportions may exhibit similar BMI scores despite differing levels of total body fat. Additionally, limitations related to cohort specificity, environmental factors, and gender disparities need to be addressed to account for the heterogeneity of SNPs and their nearby genes associated with sweet taste preference. These limitations could be mitigated by increasing the sample size and refining samples to focus on specific variables at both onset and recurrence.

## 5. Conclusions

In conclusion, this research significantly extends our understanding of the intricate genetic underpinnings of sweet taste preference and its broad impact on dietary choices and potential health outcomes. Specifically, two SNPs, rs80115239 and rs12878143, were identified as having the strongest and weakest associations with sweet taste preferences, respectively, at a genome-wide significance level. Individuals with the rs80115239 (AA) genotype exhibited a particular affinity for non-smoking, alcohol intake, and sweet taste preference compared to other phenotypes. Additionally, this phenotype was correlated with an elevated preference for sweet foods, including chocolate and cake, but a lower preference for physical activity, fruit, vegetables, coffee, and tea compared to the GG or GA genotypes in females from the NHS1 cohort. Individuals with the rs80115239 SNP, which was associated with a sweet taste preference, also showed a positive association with obesity risk in females from the NHS1 cohort but not in males from the HPFS cohort, indicating that the correlation may be sex-dependent. These findings can serve as a valuable foundation for future research aiming to elucidate the complex interplay between genetics, taste preferences, and dietary consumption, potentially paving the way for personalized nutrition strategies.

## Figures and Tables

**Table 1:** Sweet taste preference-GWAS analysis.

Lead SNPs	Adjacent Gene	CHR	BP	Minor Allele	Major Allele	MAF	OR (95% CI)	*p*-Value	Q	I^2^	CADD	RDB	eQTL (Tissue)
rs80115239	OR10J3	1	159286387	A	G	0.02	1.34 (1.20–1.49)	8.87 × 10^−8^	0.96	0	N/A	N/A	N/A
rs7549176	PARP1	1	226612399	G	A	0.12	1.18 (1.10–1.27)	4.40 × 10^−6^	0.73	0	4.944	7	Esophagus Muscularis, Cultured Fibroblasts
rs73159751	LSAMP	3	117208647	G	T	0.04	1.29 (1.16–1.44)	4.78 × 10^−6^	0.99	0	4.918	6	N/A
rs10845710	EMP1	12	13335670	A	G	0.30	0.87 (0.82–0.92)	2.50 × 10^−6^	0.43	1.05	3.972	6	N/A
rs2218442	SCAF11	12	46479401	A	T	0.10	0.85 (0.79–0.91)	2.56 × 10^−6^	0.84	0	2.383	7	Tibial Artery, Thyroid
rs12878143	RAD51B	14	69080271	C	A	0.06	0.84 (0.77–0.90)	7.38 × 10^−6^	0.85	0	3.126	7	N/A
rs58082448	SHISA6	17	11179467	A	G	0.13	1.26 (1.14–1.40)	9.80 × 10^−6^	0.70	0	N/A	N/A	N/A
rs12979148	SUGP1	18	59036788	C	T	0.40	1.16 (1.09–1.23)	6.85 × 10^−6^	0.75	0	N/A	N/A	N/A

BP, position (hg19); CADD, Combined Annotation Dependent Depletion; CHR, chromosome; CI, confidence interval; EMP1, Epithelial Membrane Protein 1; eQTL, expression quantitative trait locus; GWAS, genome-wide association study; I^2^, heterogeneity; LSAMP, Limbic System Associated Membrane Protein; MAF, minor allele frequency; NA, not available; OR, odds ratio; OR10J3, Olfactory Receptor Family 10 Subfamily J Member 3; PARP1, Poly [ADP-ribose] polymerase 1; Q, Cochran’s Q statistics; RAD51B, RAD51 Paralog B; RDB, Regulome database 2.0; SCAF11, SR-Related CTD Associated Factor 11; SHISA6, Shisa Family Member 6; SNP, single-nucleotide polymorphism; SUGP1; SURP And G-Patch Domain Containing 1.

**Table 2:** Age-standardized characteristics according to genotypes of SNP rs80115239 among US males and females in the NHS1 and HPFS.

	NHS1			HPFS		
Genotypes	GG (*n* = 10,333)	GA (*n* = 1691)	AA (*n* = 74)	GG (*n* = 6473)	GA (*n* = 1047)	AA (*n* = 35)
Age (years)	57.1 (6.8)	57 (7)	57 (7.1)	57.5 (8.7)	58.2 (8.7)	56.6 (8.7)
Caucasian (%)	99.8	99.6	97.8	94.9	95.7	95.2
BMI (kg/m^2^)	23.6 (2.7)	23.6 (2.6)	23.9 (2.6)	24.9 (2.2)	24.8 (2.3)	24.1 (2.2)
Weight (kg)	63.7 (8.5)	63.7 (8.4)	62.7 (8.3)	79.4 (9.0)	79.4 (8.9)	77.6 (9.6)
Never smokers (%)	45.0	43.2	51.8	50.6	47.2	47.9
Past smokers (%)	37.3	37.9	33.3	41.9	44.5	47.9
Current smokers (%)	17.5	18.7	14.9	7.5	8.4	4.2
Alcohol intake (g/day)	7.4 (10.7)	7.6 (11.2)	7.8 (9.0)	12.2 (15.6)	12.4 (16.0)	10.1 (11.6)
Physical activity (MET-h/week)	15.1 (20.1)	15.4 (22.9)	12.5 (14.0)	21.0 (24.1)	20.7 (24.4)	23.8 (17.3)
Total energy intake (kcal/d)	1762.0 (479.2)	1782.0 (487.6)	1735.3 (450.8)	2023.3 (605.5)	2016.0 (594.8)	1940.4 (447.6)
AHEI	46.2 (10.0)	45.8 (9.7)	46.9 (10.1)	47 (11.0)	46.5 (10.4)	43.2 (9.3)
Glycemic load	98.7 (17.2)	98.3 (17.6)	98.6 (14.7)	124.2 (25.3)	123.6 (24.4)	120.3 (18.3)
Trans fat	1.9 (0.6)	1.9 (0.6)	1.9 (0.5)	2.8 (1.1)	2.9 (1.1)	2.9 (1.2)
Total fiber	17.4 (4.8)	17.2 (4.6)	16.8 (4.0)	21.1 (6.8)	20.7 (6.3)	19.3 (4.1)
Fruit	75.1 (44.8)	74.8 (45.6)	68.3 (44.4)	69.7 (168.8)	69.8 (160.0)	48.6 (72.1)
Vegetable	85.9 (46.2)	86.2 (47.8)	74.2 (31.6)	102.2 (278.0)	96.4 (267.2)	71.5 (82.2)
Coffee	17.6 (29.9)	17.3 (30)	10.7 (21.4)	10.8 (37.4)	11.1 (38.4)	8.5 (25.8)
Tea	6.3 (5.7)	6.2 (5.8)	5.8 (4.8)	9.8 (35.6)	9.1 (34.4)	6.6 (25.5)
Sweetened beverage	37.6 (40.7)	38.6 (42.4)	31.1 (31.8)	13.9 (28.7)	13.9 (28.3)	14.6 (32.0)
Chocolate	1.5 (3.7)	1.6 (5.1)	1.6 (2.4)	3.1 (12.5)	3.2 (13.2)	3.1 (5.5)
Ice cream	1.5 (2.7)	1.6 (2.6)	1.3 (2.3)	7.7 (30.1)	9.2 (34.4)	2.5 (3.0)
Cake	2.0 (3)	2.1 (2.9)	2.2 (2.5)	6.4 (36.1)	7.0 (37.8)	4.1 (22.7)
Sleep (h/day)	7.0 (0.9)	7.1 (0.9)	7.0 (0.7)	7.1 (0.8)	7.1 (0.7)	7.2 (0.7)
Cases/person years	1824/200,769	323/33,268	24/1357	840/122,103	128/18,917	5/682
Crude incidence /100 K PY	909	971	1769	688	677	734

AHEI, alternate healthy eating index; BMI, body mass index; HPFS, Health Professionals Follow-up Study; MET, metabolic equivalent; NHS1, American Nurses’ Health Study; PY, person year. Value is not age-adjusted; data are presented as the mean (SD) for continuous variables, percentages for categorical variables, and are standardized to the age distribution of the study population.

**Table 3:** Age-standardized characteristics according to genotypes of SNP rs12878143 among US males and females in the NHS1 and HPFS.

	NHS1			HPFS		
Genotypes	TT (*n* = 8305)	CT (*n* = 3424)	CC (*n* = 369)	TT (*n* = 5326)	CT (*n* = 2026)	CC (*n* = 203)
Age (years)	57.1 (6.9)	57 (6.8)	57.8 (6.7)	57.4 (8.8)	57.9 (8.5)	58.6 (8.3)
Caucasian (%)	99.7	99.7	99.7	95.0	95.2	94.2
BMI (kg/m^2^)	23.6 (2.7)	23.6 (2.7)	23.9 (2.6)	24.8 (2.2)	24.9 (2.2)	25.1 (2.3)
Weight (kg)	63.6 (8.4)	64 (8.5)	64.3 (8.1)	79.2 (8.9)	79.7 (9.1)	80.1 (9.1)
Never smokers (%)	44.1	46.2	44.7	49.9	50.0	52.4
Past smokers (%)	37.5	37.1	38.9	42.3	42.5	41.8
Current smokers (%)	18.1	16.4	16.1	7.8	7.5	5.8
Alcohol intake (g/day)	7.4 (10.9)	7.3 (10.4)	6.9 (9.2)	12.2 (15.6)	12.5 (15.9)	11.8 (15.0)
Physical activity (MET-h/week)	15.0 (20.3)	15.7 (21.3)	14.4 (16.6)	20.8 (24.2)	21.3 (24.4)	21.6 (24.7)
Total energy intake (kcal/d)	1764.9 (482.0)	1765.4 (481.1)	1763.0 (437.3)	2016.2 (599.6)	2034.1 (610.0)	2053.2 (641.0)
AHEI	46.2 (9.9)	45.9 (10.1)	46.6 (10.2)	47 (10.9)	46.6 (10.8)	46.2 (11.8)
Glycemic load	98.7 (17.2)	98.6 (17.5)	98.1 (16.5)	123.9 (25.3)	124.6 (24.8)	123.2 (26.0)
Trans fat	1.9 (0.6)	1.9 (0.6)	1.9 (0.6)	2.9 (1.1)	2.8 (1.1)	2.9 (1.2)
Total fiber	17.4 (4.8)	17.3 (4.7)	17.4 (4.8)	21 (6.7)	21.2 (6.8)	20.9 (6.9)
Fruit	75.1 (44.5)	74.8 (46.0)	77.4 (43.3)	71.2 (170.1)	66.6 (162.5)	63.5 (161.7)
Vegetable	86.1 (46.0)	85.4 (47.3)	87.5 (46.4)	100.9 (281.2)	101.8(269.0)	101.4 (189.3)
Coffee	17.5 (30.0)	17.5 (30.0)	19.5 (30.9)	10.7 (37.2)	10.9 (37.8)	16.3 (47.0)
Tea	6.3 (5.6)	6.3 (5.7)	6.7 (6.3)	9.5 (35.1)	10 (36.1)	9.5 (35.6)
Sweetened beverage	37.6 (40.9)	37.9 (41.0)	36.5 (39.0)	13.9 (28.5)	14.7 (31.1)	10 (14.4)
Chocolate	1.6 (4.0)	1.4 (3.5)	1.6 (5.2)	3.2 (13.2)	2.8 (10.9)	4 (16.7)
Ice cream	1.5 (2.7)	1.5 (2.6)	1.6 (2.4)	7.9 (30.8)	8.1 (30.9)	5.3 (21.6)
Cake	2.0 (3.1)	2.0 (2.8)	2.0 (2.8)	6.7 (36.5)	5.9 (35.4)	5.5 (31.8)
Sleep (h/day)	7.0 (0.9)	7.0 (0.9)	7.0 (0.9)	7.1 (0.8)	7.1 (0.8)	7.2 (0.7)
Cases/person years	1485/160,988	600/67,338	86/7068	686/99,877	256/37,953	31/3872
Crude incidence /100 K PY	922	891	1217	687	675	801

AHEI, alternate healthy eating index; BMI, body mass index; HPFS, Health Professionals Follow-up Study; MET, metabolic equivalent; NHS1, American Nurses’ Health Study; PY, person year. Value is not age-adjusted; data are presented as the mean (SD) for continuous variables, percentages for categorical variables, and are standardized to the age distribution of the study population.

**Table 4:** Adjusted HR (95% CI) of obesity for genotypes of SNPs in the NHS1 and HPFS.

	Model 1 (Age-Adjusted Model) ^1^			Model 2 (Multivariate-Adjusted Model) ^2^		
Independent Variable	HR	95% CI	*p*-Value	HR	95% CI	*p*-Value
rs80115239 (AA vs. GG)						
NHS1 ^3^	1.822	1.216–2.730	0.004	1.902	1.269–2.850	0.002
HPFS ^4^	1.000	0.414–2.412	1.000	1.053	0.436–2.545	0.908
Pooled ^5^	1.642	1.137–2.370	0.008	1.716	1.188–2.479	0.004
rs80115239 (GA vs. GG)						
NHS1	1.073	0.954–1.208	0.241	1.072	0.952–1.206	0.253
HPFS	1.013	0.841–1.221	0.892	1.005	0.834–1.211	0.961
Pooled	1.056	0.955–1.167	0.288	1.052	0.952–1.163	0.321
rs12878143 (CC vs. TT)						
NHS1	1.346	1.083–1.674	0.007	1.347	1.083–1.675	0.007
HPFS	1.248	0.869–1.791	0.229	1.248	0.870–1.792	0.229
Pooled	1.319	1.095–1.590	0.004	1.320	1.095–1.591	0.004
rs12878143 (CT vs. TT)						
NHS1	0.965	0.878–1.061	0.466	0.975	0.887–1.073	0.608
HPFS	0.993	0.859–1.146	0.924	0.996	0.862–1.150	0.955
Pooled	0.974	0.899–1.054	0.508	0.982	0.907–1.063	0.646
rs9939609 (AA vs. TT) ^6^						
NHS1	1.362	1.203–1.543	<0.001	1.375	1.214–1.558	<0.001
HPFS	1.183	0.982–1.425	0.077	1.195	0.992–1.439	0.061
Pooled	1.304	1.176–1.446	<0.001	1.317	1.187–1.461	<0.001
rs9939609 (AT vs. TT)						
NHS1	1.205	1.096–1.325	<0.001	1.207	1.098–1.328	<0.001
HPFS	1.115	0.967–1.286	0.134	1.118	0.969–1.289	0.127
Pooled	1.177	1.087–1.273	<0.001	1.179	1.089–1.276	<0.001

CI, confidence interval; HPFS, Health Professionals Follow-up Study; HR, hazard ratio; NHS1, Nurses’ Health Study 1; SNP, single-nucleotide polymorphism; ^1^ Model 1 is age-adjusted; ^2^ Model 2 is adjusted for age, smoking status, alcohol consumption, physical activity, total fiber, trans-fat, and total energy intake; ^3^ Follow-up in NHS1 was from 1986 to 2014; ^4^ Follow-up in HPFS was from 1986 to 2014; ^5^ Results of two cohorts were pooled by means of inverse variance-weighted fixed-effects meta-analysis (all *p*-values for heterogeneity > 0.05); ^6^ SNP reported to be associated with the fat mass and obesity-associated protein (FTO) in the cross-sectional study.

## Data Availability

The original contributions presented in the study are included in the article; further inquiries can be directed to the corresponding authors.
